# Oral Nifidepine versus IV labetalol in severe preeclampsia: A randomized control trial

**DOI:** 10.12669/pjms.36.6.2591

**Published:** 2020

**Authors:** Tayyiba Wasim, Shazia Agha, Kanwal Saeed, Anam Riaz

**Affiliations:** 1 Dr. Tayyiba Wasim, FCPS, Department of Gynecology, Services Institute of Medical Sciences, Lahore, Pakistan; 2 Dr. Shazia Agha, FCPS, Department of Gynecology, Services Institute of Medical Sciences, Lahore, Pakistan; 3 Dr. Kanwal Saeed, FCPS-I, Department of Gynecology, Services Institute of Medical Sciences, Lahore, Pakistan; 4 Dr. Anam Riaz, FCPS-I, Department of Gynecology, Services Institute of Medical Sciences, Lahore, Pakistan

**Keywords:** Severe preeclampsia, Labetalol, Nifidepine

## Abstract

**Objective:**

To compare oral Nifidepine and IV labetalol in terms of rapidity of BP control in severe preeclampsia.

**Methods:**

All patients coming to Services Hospital from March 2017 to February 2019 with diagnosis of severe preeclampsia ≥ 24 weeks gestation were randomized to either receive Nifidepine or Labetalol. Primary outcome measure was time taken to control BP and number of doses required. Secondary outcome measures were side effects of drugs, APGAR score, NICU admission and perinatal mortality.

**Results:**

Two hundred four patients were included in trial with 102 patients in each group. Labetalol took 22.6± 13.5minutes and Nifidepine took 22.09± 11.7 minutes to achieve target BP (p>0.05). Labetalol required 2.3± 1.58 doses and Nifidepine 2.2± 1.58 doses to control BP ( p>0.05). No maternal side effects were seen in 86 (84.31%) and 92(90.19%) patients in both groups (p>0.05). Mean gestational age at birth was 34.8 ±2.73weeks in Labetalol and 35.2±2.48 weeks in Nifidepine group (p>0.05). In labetalol group, 43 (42.15%) babies had APGAR Score < 7/10 and 23(22.54%) babies required admission to NICU while in Nifidepine group 42 (41.17%) babies had Apgar score < 7/10 & 30(29.4%) babies were admitted to NICU(p>0.05). There were 21(20.5%) perinatal deaths in labetalol Group-And 19(18.6%) in Nifidepine group (p>0.05)

**Conclusion:**

Oral Nifidepine and IV labetalol are equally efficacious in controlling BP in patients with severe pre eclampsia without any significant side effects.

## INTRODUCTION

Hypertensive disorders of pregnancy complicate 4-7% of pregnancies and the second leading cause of maternal death worldwide^1^. The disease varies in spectrum from mild hypertension to preeclampsia and eclampsia. Severe preeclampsia is defined as systolic blood pressure (BP) of ≥160mmhg and diastolic BP of ≥ 110mmHg along with proteinuria of ≥ 300mg/24 hours. In addition, clinical symptoms and signs of headache, visual disturbances, epigastric pain, abnormal liver and renal function tests and thrombocytopenia may be present. If not treated, it results in pulmonary edema, cerebral hemorrhage, liver and renal failure and ultimately maternal death. As a result of placental hypo perfusion, fetal growth restriction and death may occur as well.^2^

The only effective treatment of severe preeclampsia is delivery of the fetus but immediate antihypertensive treatment is given to stabilize the patient and prevent further complications. Intravenous drugs in the form of labetalol and hydralazine have been traditionally used as first line drugs for control of severe hypertension in pregnancy. Although adverse effects of drugs have been reported but both are effective in controlling BP in severe pre eclampsia. Nifidepine is a calcium channel blocker with advantages of oral administration. Worldwide trials comparing Nifidepine, hydralazine and labetalol in hypertensive emergency have been conducted with variable results regarding their efficacy in controlling BP and fetal effects.^3^-^5^ Duley et al in Cochrane data base review reported lack of evidence regarding choice of antihypertensive.^6^ Recent systematic review has declared all three drugs equally effective in controlling BP in hypertensive emergencies in pregnant women.^7^ NICE guidelines recommend Labetalol, hydralazine and Nifidepine as first line anti-hypertensive in severe pre eclampsia.^8^

Pakistan has high burden of hypertensive disease in pregnancy. Eclampsia is second major cause of maternal death and claims 2000 maternal lives per year.^9^ IV hydralazine and labetalol are used for severe pre eclampsia in Pakistan depending on their availability. Nifidepine can be a good alternative in our set up, as it is cheap and can be given orally in rural settings of basic health units and tehsil head quarter hospitals. No trial comparing IV labetalol and Nifidepine has been reported from Pakistan. There are no guidelines for management of preeclampsia in Pakistan. We planned this trial with the aim to compare oral nifedipine with IV labetalol for BP control so that recommendations for choice of drugs in severe hypertension can be made.

## METHODS

This open label randomized control study was conducted in the Department of Obstetrics & Gynecology, Services Institute of Medical Sciences, Services Hospital Lahore from March 2017 to February 2019. Services Hospital Lahore is a tertiary care hospital attached to public sector medical college, catering the needs of a large population. Ethical approval for the study was taken by the Institutional Review Board (IRB) SIMS, Services Hospital Lahore on February 16, 2017 number IRB/2017/306/SIMS and Trial Registration no: NCT03325348.

All pregnant patients’ ≥28 weeks of gestation diagnosed with severe pre eclampsia as defined by systolic BP of ≥160mmHg or diastolic BP of ≥110mmHg with proteinuria and alive baby were admitted and offered to enrol in the trial after written informed consent. Women with history of chronic hypertension without proteinuria, with heart rhythm abnormalities, asthma, anomalous baby and intrauterine death were excluded from the trial. Detailed history regarding booking, drugs for hypertension, symptoms of headache, blurring of vision, dizziness and epigastric pain was taken. Their relevant investigations of blood group, complete blood count, urine complete examination, serum uric acid, liver function tests, clotting profile were sent. Ultrasound was done to see fetal wellbeing and biometry along with liquor volume. The patients were randomized to two groups based on computer generated random numbers, 102 patients in each Group-After sample size calculation. The patients in Group-A were given intravenous labetalol injection (in an escalating dose regimen of 20, 40, 80, 80 and 80 mg) and a mint tablet every 15 minutes. Patients in Group-B were given Nifidepine (10mg tablet, orally every 15 minutes up to five doses) and intravenous placebo saline injection. BP was recorded every 15 minutes until the target blood pressure of ≤150/100mmHg was achieved. Fetal heart rate monitoring was done every 15 minutes throughout administration. If BP was not controlled in one hour, other antihypertensive drugs were given. Similarly, after successful BP control, routine antihypertensive therapy was started two hours after last study drug administration. Corticosteroid injection were given for lung maturity to all participants less than 36 weeks duration. Depending on fetus and maternal condition, delivery was planned according to gestational age and bishop score.

Primary outcome measure was time taken to control the BP and number of doses of drugs required. Secondary outcome measure were maternal complications of placental abruption, HELLP and eclampsia. Neonatal outcome was measured in form of Apgar score at five minutes, admission to NICU and perinatal death. Side effects of both drugs were also noted. Data was entered and analyzed using SPSS version 23. The comparison between qualitative variables was done by using chi square test or fisher Exact test where appropriate. All P-values were two tailed and p-value of 0.05 or less was considered significant.

### Sample Size

Sample size was calculated by using WHO statistical software (S.size). Assumption for sample size estimation was the hypothesis test for difference of two proportions (two sided test). The estimated sample size is sufficient to detect the difference of 20% among the two groups at significance level 5% and power of study 90%. The estimated sample size was 102 for each group.

## RESULTS

Total 204 patients were recruited in this randomized controlled study. Group-A with 102 patients was given IV labetalol and Group-B with 102 patients was given oral Nifidepine. Patient characteristics are shown in Table-I. Age of patients was 28.1±4.37 years and 24.6±4.65 in both groups respectively while 71 (69.6%) and 62 (60.78%) in each group were primigravida (p=0.217). In Group-A, 66(64.70%) patients were booked and in Group-B 70(68.62%) patients were booked (p>0.05).

**Table-I:** Maternal characteristics.

Maternal Characteristics		Group-A Labetalol (n=102)	Group-B Nifidepine (n=102)	P value
Maternal Age(years) Mean		28.15 ±4.372	24.65±4.652	0.217
Primigravida		71 (69.60%)	62 (60.78%)	0.186
Multigravida		31 (30.39%)	40 (39.21%)	0.186
Socioeconomic Status	Poor	24 (23.52%)	31 (30.39%)	0.342
	Middle	55 (53.92%)	55 (53.92%)	
	High	23 (22.54%)	16 (15.68%)	
Gestational age Weeks		34.83±2.736	35.26±2.485	0.292
Booking status	Booked	66 (64.70%)	70 (68.62%)	0.552
	Unbooked	36 (35.29%)	32 (31.37%)	

Regarding primary outcome of the study, that is time taken to achieve blood pressure control as shown in Table-II, average time taken was 22.69±13.5 minutes in Group-A & 22.09± 11.7 minutes in Group-B (p=0.110). Mean doses required in each group were 2.36±1.58 doses in Group-A and 2.28±1.58 doses in Group-B (p=0.183). All patients had their BP controlled in one hour. No maternal side effects were seen in 86 (84.31%) patients of Group-A and 92(90.19%) patients in Group-B (p=0.521). No maternal complications were seen in 93 (91.17%) and 91 (89.2%) of patients in both groups respectively (p=0.561). Eclampsia and placental abruption was seen in 4(3.92%) patients each in Labetalol Group-A and 03 patients developed eclampsia and 7(6.86%) patients had placental abruption in Group-B.

**Table-II:** Fetomaternal Outcome.

Fetomaternal OUTCOME		Group-A Labetalol	Group-B Nifidepine	P value
Time (minutes) taken to achieve blood pressure Mean		22.69±13.57	22.09±11.74	0.110
Total antihypertensive doses to achieve blood pressure Mean		2.36±1.581	2.28±1.581	0.183
Mode of delivery	Vaginal	44 (43.13%)	49 (48.03%)	0.482
	Caesarean	58 (56.86%)	53 (51.96%)
Birth Weight(kg) Mean		2.28±0.411	2.38±0.389	0.182
Gender of Baby	Male	62 (60.78%)	49 (48.03%)	0.068
	Female	40 (39.21%)	53 (51.96%)	
NICU admission	Yes	23 (22.54%)	30 (29.41%)	0.264
	No	79 (77.45%)	72 (70.58%)	
APGAR score	<7/10	43 (42.15%)	42 (41.17%)	0.887
	>7/10	59 (57.84%)	60 (58.82%)	
Neonatal Outcome	Live Births	81 (79.41%)	83 (81.37%)	0.328
	Stillbirths	03(2.95%)	05 (4.9%)	
	Neonatal Death	18 (17.64%)	14 (13.72%)	
Maternal Side effects	No side effects	86 (84.31%)	92 (90.19%)	0.521
	Palpitation	10 (9.80%)	8 (7.84%)	
	Headache	4 (3.92%)	1 (0.98%)	
	Dizziness	1 (0.98%)	0	
	Hypotension	1 (0.98%)	1 (0.98%)	
Maternal Complications	No complication	93 (91.17%)	91 (89.21%)	0.561
	Eclampsia	4 (3.92%)	3 (2.94%)	
	Placental Abruption	4 (3.92%)	7 (6.86%)	
	HELLP	0	1 (0.98%)	
	DIC	1 (0.98%)	0	

Mean gestational age at birth was 34.8±2.73 weeks in Group-A and 35.2±2.48 weeks in Group-B (p=0.292) with mean birth weight of babies 2.2±0.41 kg and 2.3±0.38 kg respectively in Group-A and B. In Labetalol Group, 43 (42.15%) babies had APGAR Score < 7/10 and 23(22.54%) babies required admission to NICU. In Nifidepine group 42 (41.17%) babies had Apgar score < 7/10 & 30(29.4%) babies were admitted to NICU (p>0.05). There were 81 (79.5%) live births, 3 (2.9%) still births & 18(17.6%) early neonatal deaths while in Nifidepine group, there were 83 (81.3%) live births, 5(4.9%) still births & 14 (13.7%) neonatal deaths (p=0.328).

## DISCUSSION

Mean age of our patients was comparable in both groups (28.1±4.37 years and 24.6±4.65 years (p>0.05). 69.6% and 60.7% patients in both groups were primigravida, as is the case worldwide that majority are primigravida.^3^-^7^

In our study, labetalol and Nifidepine were found to be equally effective in controlling BP in pregnant women with severe preeclampsia taking mean time of 22.69±13.5 and 22.09±11.7 minutes each(p>0.05). Number of doses required were not statistically different in both groups as mean dose to control BP was 2.36±1.5 doses in labetalol Group-And 2.28±1.58 doses in Nifidepine group(p>0.05). Similar trials conducted by Raheem et al, Shekhar et al, Anjuman et al and Yogita et al showed that BP control was controlled significantly earlier in patients who were given Nifedipine as compared to labetalol and they required less number of doses.^7^,^10^-^12^ Lakhshami et al declared labetalol superior regarding earlier control of BP while Shi DD et al reported both achieved BP control at same time with two doses in both groups.^13^,^14^ The difference results in various trials may be related to the smaller number of participants in each group. The results of our study showing oral nifedipine equally efficacious in controlling BP in comparison to IV drug has important implications in our set up where cost, availability and administration of IV drugs is a big issue Orally used drug will have a wider use in communities, basic health units and LHVs which receive the major burden of disease.

The side effects profile of the patients was comparable and 84.3% patients of labetalol Group-And 90.1% patients in Nifidepine group did not experience any side effects(p>0.05). Only few experienced palpitations, headache and hypotension which was well tolerated. Another study from Lahore comparing Nifidepine to hydralazine has also shown minimal side effects with nifedipine.^15^ Raheem et al and Anjuman et al reported significantly increased maternal heart rate in patients who received Nifidepine but this was not seen in our study. Shekar et al in their review have reported significantly reduced side effects in patients who were given nifidepine.^16^


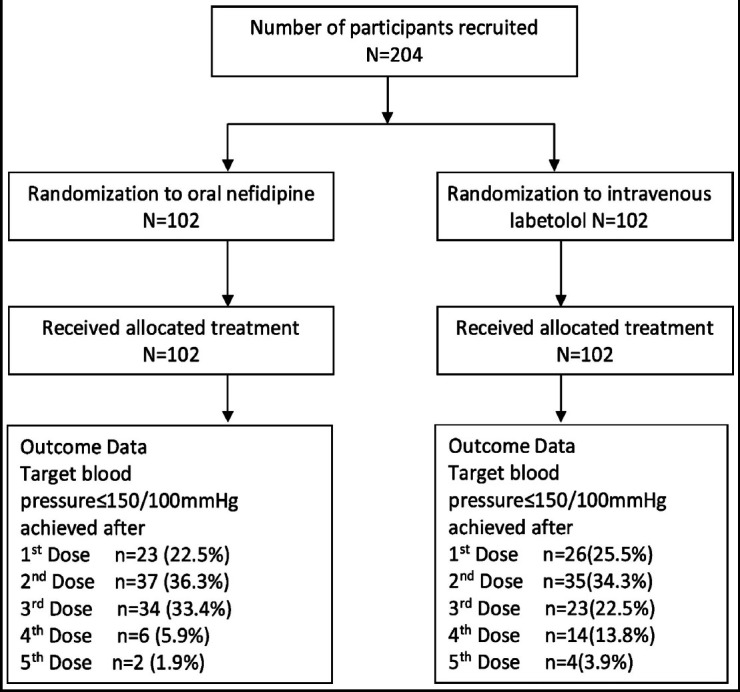


In this study, average gestation was 34.8±2.73 weeks in labetalol Group-And 35.2±2.48 weeks in patients given Nifidepine with average birth weight of 2.2±0.41kg and 2.3±0.38 kg respectively (p>0.05) This is in contrast to studies reported by Raheem et al and Yogita et al where majority babies delivered at gestational age of 37-39 weeks.^5^,^12^ The reason may be that quite a lot of our patients were unbooked and were not taking any antenatal care. Regular antenatal care has key role to play in BP control and hence prolongation of pregnancy.

APGAR score<7 at five minutes was seen in 43.15% and 41.17% patients in labetalol and Nifidepine groups respectively. Although more patients (29.4%) on Nifidepine required NICU admissions as compared to 22.5% patients in labetalol Group-But it was not statistically significant(p>0.05). Li QQ in meta-analysis reported better BP control with Nifidepine but no difference in APGAR score and perinatal outcome.^16^ Yogita et al and Anjuman et al reported significantly less number of NICU admissions in patients who were given Nifidipine.^11^,^12^ Shekar et al reported that APGAR score and NICU admissions were comparable in both groups but risk of neonatal death was decreased in patients who were given Nifidepine.^17^ Our study showed 21(20.5%) perinatal deaths in labetalol group with three stillbirths and 18 early neonatal deaths while patients in Nifidepine group had 19(18.6%) perinatal deaths with five stillbirths and 14 early neonatal deaths which is statistically insignificant (p>0.05). The reason for stillbirths was abruption and severe IUGR.

Prematurity and growth restriction have a major role in adverse perinatal outcome in this study. NICU in government hospitals is overburdened with overwhelming number of patients and poor resources lead to increased chances of early neonatal death in premature babies. Increasing burden of perinatal mortality for severe hypertension is reported from other developing countries as well.^18^ Perinatal mortality rates are comparable in both groups in our study. Firoz T in meta-analysis showed similar success in control of BP with both drugs but better neonatal outcome in patients receiving nifedipine.^19^

## CONCLUSION

Oral Nifidepine and IV labetalol are equally efficacious in controlling BP in patients with severe pre eclampsia without any significant side effects. Neonatal outcome in terms of NICU admission and perinatal mortality is also comparable. Nifidepine can be recommended as first line drug for control of severe hypertension due to ease of oral administration, cost effectiveness and easy availability.

### Authors’ Contribution:

**TW:** Conceptualized and designed the study, reviewed the manuscript and approved the final version. She is responsible for accuracy and integrity of work.

**SA:** contributed to maintenance of data base and initial writing of script.

**KS & AR:** did the data entry and statistical analysis.
